# Polyneuropathy in Kidney Transplant Recipients: Accuracy of a New Clinical Diagnostic Scoring System

**DOI:** 10.1111/jns.70058

**Published:** 2025-09-07

**Authors:** Svea Nolte, Naser B. N. Shehab, Stefan P. Berger, Celina Oldag, Ilja M. Nolte, Bianca T. A. de Greef, Fiete Lange, Marco van Londen, Catharina G. Faber, Stephan J. L. Bakker, Pieter A. van Doorn, Harmen R. Moes, Gea Drost

**Affiliations:** ^1^ Department of Neurology University Medical Center Groningen, University of Groningen Groningen the Netherlands; ^2^ Department of Internal Medicine, Division of Nephrology University Medical Center Groningen, University of Groningen Groningen the Netherlands; ^3^ Department of Epidemiology University of Groningen, University Medical Center Groningen, University of Groningen Groningen the Netherlands; ^4^ Department of Neurology, Mental Health and Neuroscience (HHeNS) Research Institute Maastricht University Medical Center Maastricht the Netherlands; ^5^ Department of Neurology Erasmus University Medical Center Rotterdam the Netherlands; ^6^ Department of Neurosurgery University Medical Center Groningen, University of Groningen Groningen the Netherlands

**Keywords:** kidney transplantation, neurological diagnostic techniques, polyneuropathy

## Abstract

**Background and Aims:**

Polyneuropathy is highly prevalent among kidney transplant recipients (KTR), underscoring the need for an accurate yet easy‐to‐perform diagnostic method to improve understanding and enable early identification of treatable cases.

**Methods:**

This study included KTR at least 12 months post‐transplant at the University Medical Centre Groningen, the Netherlands. An expert panel assessed polyneuropathy through a structured neurological examination, quantitative sensory testing, and nerve conduction studies. The modified Toronto Clinical Neuropathy Score (mTCNS) was obtained from all participants. Logistic regression analyses with Firth penalization validated the mTCNS components. A new model, the Kidney Transplant Neuropathy Score (KTNS), was developed through stepwise elimination. Diagnostic performance was evaluated with bootstrapped metrics and ROC curve analyses.

**Results:**

Among 160 KTR, 91 (57%) were diagnosed with polyneuropathy. All 10 mTCNS components were univariably associated with polyneuropathy; numbness (OR = 4.9 [1.8–18.0]), tingling (OR = 2.5 [1.2–5.9]), impaired nociception (OR = 1.5 [1.1–2.2]), and reduced vibration perception (OR = 1.5 [1.0–2.4]) remained independently associated in multivariable analysis. The mTCNS achieved an area under the curve (AUC) in ROC analysis of 0.83 [0.76–0.89]. Two KTNS were derived: the KTNS_Basic_, including history of numbness, tingling in the feet, and pinprick and vibration perception testing (AUC–ROC: 0.85 [0.79–0.90]); and the KTNS_Advanced_, replacing vibration perception with Achilles and patellar deep tendon reflex testing (AUC–ROC: 0.90 [0.85–0.94]).

**Interpretation:**

The mTCNS is a valid diagnostic tool for polyneuropathy in KTR. The KTNS offers a simplified alternative based on key symptoms and sensory tests, with reflex testing included in the KTNS_Advanced_ for settings with neurological expertise.

**Trial Registration:**

ClinicalTrials.gov identifier: NCT04664426

## 1 Introduction

Polyneuropathy has been shown to be highly prevalent in kidney transplant recipients (KTR) [1]. It is known that patients are at high risk of developing polyneuropathy prior to kidney transplantation. The prior state of chronic kidney disease, with diabetes mellitus being a potential cause of kidney failure, is supposed to be a major contributor [2, 3]. After kidney transplantation, the use of immunosuppressive medication may result in further development of polyneuropathic symptoms and signs [4, 5]. In a previous study of our research group, we demonstrated that polyneuropathy is significantly underdiagnosed in KTR inclinical practice, likely due to the complexity of the diagnosis [1]. To gain a better understanding of the pathophysiology, it is necessary to diagnose and stage polyneuropathy in an easy and convenient way in KTR. Moreover, early diagnosis is crucial in clinical practice because treatment can prevent further deterioration of neuropathic symptoms and signs or even reverse neurological deficits in certain types of polyneuropathy [6].

The diagnosis of polyneuropathy requires a systematic approach combining clinical assessment and confirmatory testing. Although the initial recognition often relies on characteristic symptoms and signs, the formal diagnosis traditionally depends on specialized neurophysiological testing such as nerve conduction studies (NCS) or quantitative sensory testing (QST) [7, 8]. These tests not only confirm the presence of polyneuropathy but also help characterize the type (small vs. large fiber) and distribution of nerve involvement. However, such specialized testing is time‐consuming, relatively expensive, and requires specific expertise. To address these practical limitations, various clinical scoring systems have been developed that standardize and quantify the assessment of polyneuropathic symptoms and signs [9, 10]. Although these scoring systems cannot replace neurophysiological testing for detailed characterization of polyneuropathy, they have proven valuable as screening tools to identify patients who require further diagnostic evaluation. The Toronto Clinical Neuropathy Score (TCNS) was initially designed for the detection of diabetic sensorimotor polyneuropathy, but its usefulness extends beyond diabetic polyneuropathy [9, 11]. Eventually, the TCNS was modified (into the mTCNS) to better capture a range of simple sensory tests representative of early‐stage dysfunction. Reflex testing was removed from the TCNS because of its high inter‐rater variability, enhancing the reliability and consistency of the assessment [12]. The modified Toronto Clinical Neuropathy Score (mTCNS) was demonstrated to maintain an acceptable correlation with the precursor score, the TCNS [12].

Given the high prevalence of polyneuropathy in KTR and their regular follow‐up in transplant clinics, there is a particular need for a simple screening tool that can be used by transplant physicians who may not have extensive neurological expertise. Additionally, such a tool would facilitate large‐scale research on polyneuropathy in this population, potentially leading to a better understanding of its pathophysiology and treatment options. Therefore, the goal of the current study was to validate the mTCNS in a cohort of KTR and to investigate whether the performance of the mTCNS can be enhanced.

## 2 Materials and Methods

### 2.1 Population and Study Design

The current study is part of the SENS (Sensory Neuropathy Scores) study at the University Medical Center Groningen (UMCG), the Netherlands (ClinicalTrials.gov identifier). This study involved prospective data collection from KTR, at least 12 months post‐transplantation, who participated in the study between December 2021 and May 2023. KTR were recruited through simple random sampling after participating in the TransplantLines Biobank and Cohort Study at the UMCG [13]. The following inclusion criteria were followed: at least 18 years of age, able to understand the Dutch language, and capable of following the instructions during the neurological testing. KTR were excluded from participation if one of the following conditions applied: bilateral amputation of upper or lower limbs, bilateral limb injury (including an arteriovenous dialysis shunt), bilateral metal implants in the limbs, pacemaker or implantable cardioverter defibrillator, previous diagnosis with a mononeuropathy including carpal tunnel syndrome, pregnancy, restart of dialysis due to transplant failure, previous treatment with chemotherapy, or use of mind‐altering drugs. Demographic and clinical data were extracted from medical records for all included patients. During a single study visit at the outpatient clinic of the UMCG, clinical examinations according to the study protocol were performed. The study was approved by the local Medical Ethical committee (METc 2020/438). All procedures were in accordance with the Declaration of Helsinki and the Declaration of Istanbul. The manuscript was prepared following the Standards for Reporting of Diagnostic Accuracy Studies (STARD) statement [14].

### 2.2 Modified Toronto Clinical Neuropathy Score

Examination of polyneuropathic symptoms and signs consisted of two components, namely history of symptoms and sensory testing. For the symptom scores, participants were asked for each of the following symptoms separately if they were present in the feet: neuropathic pain, numbness, tingling sensation, muscle weakness, and loss of sensation leading to ataxia. They were also asked if any of the symptoms were present in the hands for one additional symptom score. If symptoms were absent, participants scored a 0. If symptoms were present, participants were asked to what level these interfered with their sense of well‐being and/or activities of daily living. If they interfered with activities of daily living, participants scored a 3; if they interfered with well‐being but not with activities of daily living, participants scored a 2; and if neither was the case, participants scored a 1.

**FIGURE A1:**
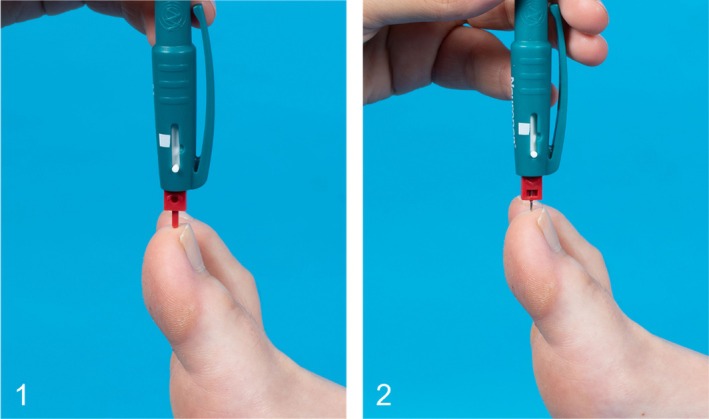
Assessment of nociception using the pinprick method at the level of the big toe.

**FIGURE A2:**
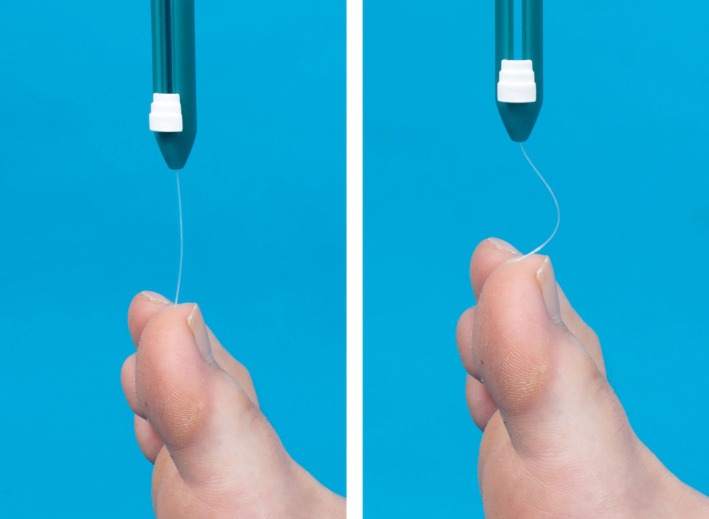
Assessment of light touch perception using the monofilament at the level of the big toe.

**FIGURE A3:**
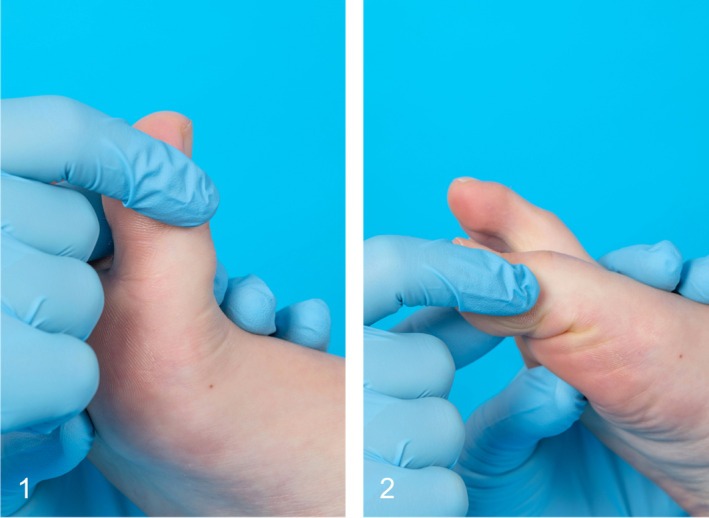
Assessment of proprioception at the level of the big toe.

**FIGURE A4:**
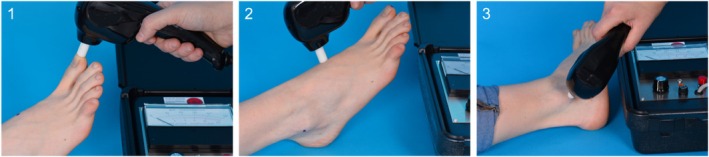
Assessment of vibration perception using a biothesiometer.

All sensory tests were performed bilaterally at three different testing sites: big toe, instep, and the shin. Nociception and light touch perception were tested with a neuropen (Neuropen, Owen Mumford Ltd., Oxfordshire, United Kingdom). Nociception was assessed using pinprick testing, which involved distinguishing the sharp end of the neurotip from the blunt end. To measure light touch perception, participants were asked if they could feel a bowed 10‐g monofilament on their skin. Proprioception was tested by moving the big toe toward the nose or away from it. Vibration perception was tested using a handheld biothesiometer (Bio‐thesiometer, Bio Medical Instrument Co., Ohio) with an applicator button that vibrates at a frequency of 120 Hz and a vibration amplitude ranging from 0 to 25.5 μm of motion. Instead of the shin, vibration perception was tested on the lateral malleoli as the third level. Normal values were provided by a previous study that was carried out in the same population of healthy subjects, screened for a possible kidney donation [15]. Sensory test scores were graded as 1 if sensation was reduced at both toes only, as a 2 if it was additionally reduced on both sides at the instep, and 3 if it was further reduced to a level above the lateral malleoli on both sides. Otherwise, they were graded as 0. The testing of temperature sense was omitted for logistic reasons. For a more detailed description of the testing protocol, see the Appendix A and Figures A1, A2, A3, A4.

### 2.3 Additional Neurological Assessment

In addition to the mTCNS, the original components of the TCNS Achilles and patellar tendon reflexes were assessed bilaterally and scored as 0 if normal, 1 if reduced, and 2 if absent. In cases of discrepancies between the two sides, the superior side was taken as the reference.

Additionally, proximal and distal muscular strength of the arms and legs was evaluated. The strength of the foot dorsiflexors, hip flexors, elbow flexors, and three‐point grip was tested with a handheld dynamometer (CIT Technics, Haren, the Netherlands). The cutoffs for strength assessment using the handheld dynamometry applied in this study were based on those reported by Van der Ploeg et al. [16] Muscle weakness was categorized as distal muscle weakness (weakness of foot dorsiflexors or three‐point grip), proximal muscle weakness (weakness of hip flexors or elbow flexors), or overall muscle weakness (weakness of foot dorsiflexors and hip flexors, or three‐point grip and elbow flexors) for either lower or upper limbs, respectively.

### 2.4 Polyneuropathy

To identify large fiber deficits, NCS were performed using a Synergy EDX (Cephalon, Nørresundby, Denmark) with surface stimulation following standard techniques. Sensory nerve conduction was performed unilaterally of the ulnar and sural nerves, and motor nerve conduction of the ulnar, peroneal, and tibial nerves. Furthermore, a Soleus H‐reflex was recorded. During the examinations, skin temperature of hands and feet was closely monitored (above 31°C). Sensory nerve action potential (SNAP) and compound muscle action potential (CMAP) amplitudes, nerve conduction velocity, and, in the case of motor nerves, distal motor latency were determined. SNAP and CMAP amplitudes were measured from baseline to negative peak. For peripheral motor and sensory NCS, normal values of our clinical neurophysiology department were used. For the H‐reflex, normal values for the latency of Schimsheimer et al. [17] were used. To assess small fiber function, QST was performed unilaterally on the same side of the body as the NCS. The QST protocol included warm and cold temperature threshold testing according to the method of levels, which was performed using a TSA‐II‐NeuroSensory Analyzer (Medoc, Ramat Yishay, Israel) [18]. Testing was performed first at the foot dorsum (S1 dermatome) and subsequently on the thenar eminence (C6 dermatome) with the thermode (surface 30 × 30 mm) being attached to the skin by means of elastic Velcro tape. The thermode baseline temperature was 32°C. Skin temperature was controlled before the testing and ensured to be above 31°C. The obtained thresholds were compared with published normal values [19]. QST and NCS were carried out by neurophysiology technologists under the supervision of a neurologist. Both the neurophysiology technologists and the neurologist were blinded to the results of the mTCNS and additional neurological assessments, and vice versa.

All participants were assigned to a diagnostic class of polyneuropathy using a staged approach, as described in more detail in Appendix B. An expert panel consisting of a neuromuscular specialist and clinical neurophysiologist (G.D.), a neurophysiology specialist (F.L.) and a neurologist (H.R.M.) initially classified all patients through an individual assessment. Any discrepancies in the diagnostic classifications were subsequently discussed in a plenary session, where a final decision on the presence of polyneuropathy was assigned to each patient. For the analyses in the current study, the classifications “no” and “possible” polyneuropathy were combined into the category no polyneuropathy, and the classifications “probable” large fiber polyneuropathy, “probable” small fiber neuropathy, and “definite” large fiber polyneuropathy were combined into the category polyneuropathy.

### 2.5 Statistical Analyses

Continuous variables with a normal distribution were described as mean ± standard deviation or as median [interquartile range] in case of a skewed distribution. Histograms and Q/Q plots were used to assess the distribution of continuous variables. Categorical variables were displayed as frequencies with percentages.

Univariable logistic regression models with Firth penalization were initially performed to assess the association between components of the mTCNS and the presence of polyneuropathy. Firth‐penalized logistic regression addressed quasi‐complete separation in the data by reducing bias and enhancing parameter estimation [20]. The components of the mTCNS were treated as ordinal variables. Odds ratios (ORs) with 95% confidence intervals (CIs) were calculated for each component individually [21]. Subsequently, a multivariable logistic regression model with Firth penalization including all mTCNS components was constructed to evaluate their independent associations with polyneuropathy. A backward stepwise elimination procedure based on the likelihood ratio test (LRT) was employed to derive a reduced, parsimonious model by recursively excluding predictors whose exclusion did not significantly decrease model fit. This new reduced model is presented as the Kidney Transplant Neuropathy Score (KTNS). All CIs and tests in the Firth logistic regression models were based on the profile penalized log likelihood.

In sensitivity analyses, the described method was repeated, including the additional neurological assessments (i.e., testing of Achilles tendon reflex, patellar tendon reflex, and muscle strength in lower and upper limbs). Achilles and patellar tendon reflexes were handled as ordinal variables, whereas muscle strength was simplified to a dichotomous variable (normal vs. abnormal muscle strength).

Receiver operating characteristic (ROC) curves for the mTCNS and KTNS were generated to evaluate diagnostic performance based on the aggregated component scores. Areas under the curve (AUCs) with 95% CIs were calculated, as implemented by the pROC package in R [22]. To formally compare discrimination between the three tools (i.e., mTCNS, KTNS_Basic_, KTNS_Advanced_), we performed paired sign‐flip permutation tests on the differences in AUC between each pair of tools. Under the null hypothesis that the respective two tools are equally informative, each patient's pair of scores is exchangeable. Accordingly, within each patient, we randomly swapped scores with a probability of 0.5 across *B* = 5000 permutations (labels fixed) to approximate the null distribution for ΔAUC under exchangeability. Two‐sided *p* values were computed using the plus‐one correction: $p=\left(1+\sum |\Delta_{\text{perm}}|\geq |\Delta_{\mathrm{obs}}|\right)/\left(B+1\right)$. To account for multiple testing across the three pairwise comparisons, we applied Holm's correction. Furthermore, calibration curves were produced to assess the agreement between observed and predicted probabilities of polyneuropathy based on the different diagnostic models. A decision curve analysis was performed to show the net benefit (the difference between the number of true‐positive polyneuropathy diagnoses and the number of false‐positive diagnoses) of the diagnostic models.

To investigate the impact of different cutoff values on the performance of the mTCNS and KTNS in terms of sensitivity and specificity for detecting polyneuropathy, we evaluated a range of cutoff points for each scale. Performance metrics and their variability were estimated at each fixed cutoff value using a bootstrapping approach with 1000 resamples for internal validation [23]. In each bootstrap iteration, participants were sampled with replacement to create new datasets, and sensitivity and specificity were calculated to simulate the sampling distributions of these metrics. Point estimates of the metrics at each cutoff were determined as the medians of the bootstrapped sampling distributions. The 95% CIs were obtained using the percentile method based on the 2.5th and 97.5th percentiles. Medians and percentile‐based CIs were chosen to avoid making distributional assumptions. Optimal cutoff scores were determined according to the Euclidean index, which represents the distance from the coordinates (0, 1) in the left corner of the ROC curve [24].

SPSS version 28 for Windows (IBM, Armonk, USA) and R Statistical Software version 4.0.5 (R Foundation for Statistical Computing, Vienna, Austria) were used for statistical analyses. *p* values below 0.05 were considered statistically significant.

## 3 Results

### 3.1 Study Population

**FIGURE 1:**
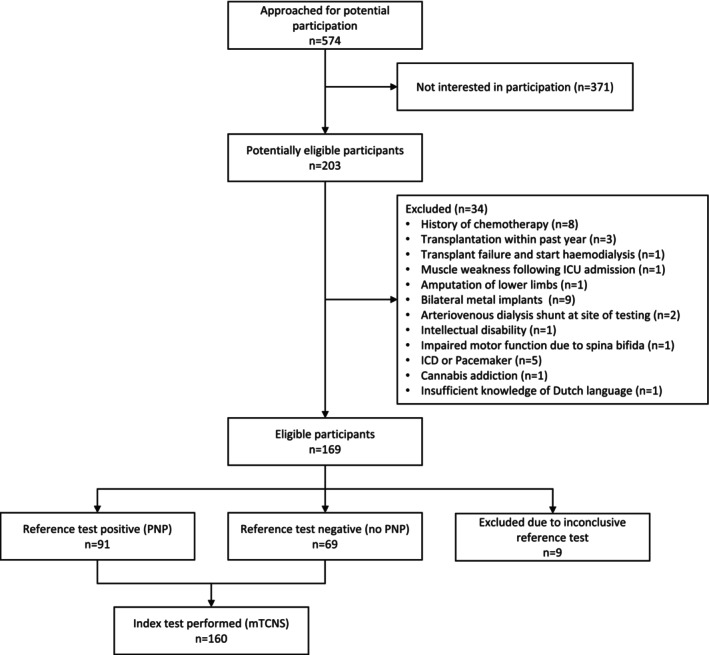
STARD flow diagram. PNP, polyneuropathy, reference test: polyneuropathy classification of expert panel; index test: modified Toronto Clinical Neuropathy Score (mTCNS).

At the start of the study inclusion, 574 KTR were approached for potential participation in the SENS study. Of these, 371 KTR were not interested in participating. A total of 203 KTR were ultimately assessed for eligibility, of whom 34 KTR were excluded based on the exclusion criteria. Subsequently, 169 KTR underwent the structured neurological evaluation, upon which the expert panel classified 91 KTR as having polyneuropathy, 69 KTR as having no polyneuropathy, and 9 KTR were excluded from the study because of insufficient information to allow for accurate classification. This rendered 160 KTR eligible for analyses to validate the mTCNS (Figure 1).

The cohort's representativeness was assessed by comparing baseline characteristics with those of 1473 KTR from the TransplantLines cohort (Appendix C). A slight but statistically significant difference in mean age was observed between the two groups (SENS cohort: 59.8 ± 11.6 years vs. TransplantLines cohort: 57.8 ± 14.0 years, *p* = 0.04). No other differences in baseline characteristics were identified.

Table 1 summarizes the characteristics of the study population. The mean age was 59.8 ± 11.6 years (range: 23–79 years), with 107 (67%) KTR being male. The median time since transplantation was 6.1 [3.9–13.1] years. A comparison of baseline characteristics between KTR with and without polyneuropathy is presented in Appendix D. Statistically significant differences were observed in age (*p* < 0.001), sex (*p* = 0.001), height (*p* = 0.002), weight (*p* < 0.001), and smoking status (*p* = 0.04).

**TABLE 1:** Baseline characteristics.

	KTR
*N*	160
Age, years	59.8 ± 11.6
Sex, *n* (%) male	107 (66.9)
Height, cm	176.2 ± 8.8
Weight, kg	81.3 ± 14.3
Time since transplantation, years	6.1 [3.9–13.1]
Primary disease, *n* (%)	
Unknown	26 (16.3)
Glomerulonephritis	45 (28.1)
Pyelonephritis	13 (8.1)
Cystic kidney disease	37 (23.1)
Other congenital/hereditary disease	5 (3.1)
Hypertensive nephropathy	14 (8.8)
Diabetic nephropathy	11 (6.9)
Other multisystem diseases	7 (4.4)
Other	2 (1.3)
Diabetes, *n* (%)	36 (22.5)
Type 1 diabetes	5 (3.1)
Type 2 diabetes	12 (7.5)
PTDM	19 (11.9)
Pre‐emptive transplantation, *n* (%)	67 (41.9)
Dialysis prior to transplantation^a^, *n* (%)	93 (58.1)
Hemodialysis	55 (34.4)
Peritoneal dialysis	46 (28.8)
Dialysis vintage, months	25 [9–42]
Type of transplantation	
Kidney	156 (97.5)
Combined kidney and pancreas	4 (2.5)
Donor type, *n* (%)	
Living donor	97 (60.6)
Deceased donor	63 (39.4)
Donation after brain death	33 (20.6)
Donation after circulatory death	29 (18.1)
Alcohol intake^1^, units/week	1.2 [0–6.3]
Smoking status^2^, *n* (%)	
Never	79 (54.5)
Ex‐smoker	57 (39.3)
Current	9 (6.2)
Immunosuppression	
Calcineurin inhibitor use, *n* (%)	137 (85.6)
Tacrolimus use, *n* (%)	118 (73.8)
Tacrolimus dosage, mg	3.0 [2.0–4.0]
Whole blood concentration tacrolimus, μg/L	5.1 ± 1.4
Cyclosporine use, *n* (%)	19 (11.9)
Cyclosporine dosage, mg	160.5 ± 63.6
Whole blood concentration cyclosporine, μg/L	76.0 [54.5–97.5]
Prednisolone use, *n* (%)	155 (96.9)
Third immunosuppressive	
MMF, *n* (%)	121 (75.6)
Everolimus, *n* (%)	10 (6.3)
Azathioprine, *n* (%)	7 (4.4)
Other, *n* (%)	3 (1.9)

*Note:* Missing data (*n*): ^1^12, ^2^15.

Abbreviations: KTR, kidney transplant recipients, MMF, mycophenolate mofetil; PTDM, post‐transplant diabetes mellitus.

^a^ Eight KTR received both hemodialysis and peritoneal dialysis prior to transplantation.

Polyneuropathy was present in 26 (72.2%) KTR with diabetes and in 65 (52.4%) KTR without diabetes.

### 3.2 Components of the mTCNS

**FIGURE 2:**
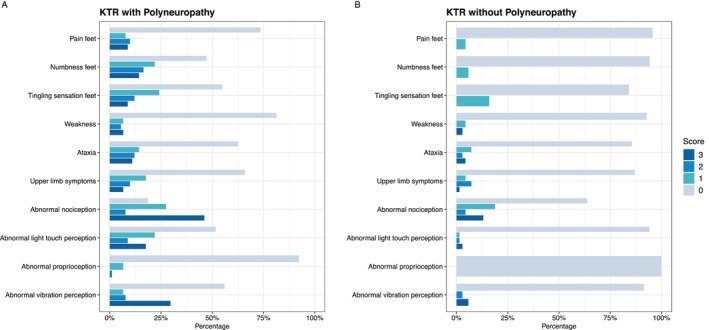
Frequency of symptoms and signs of the mTCNS in KTR. The bar plot is indicating the frequency of symptoms and signs in KTR with polyneuropathy (A) and KTR without polyneuropathy (B). The score can be interpreted as follows: 0: Absent (symptoms), normal (testing) in grey. 1: Present but no interference with sense of well‐being or activities of daily living (symptoms), reduced at toes only (signs) in green. 2: Present, interferes with sense of well‐being but not with activities of daily living (symptoms), reduced to a level above the toes but only up to the ankles (signs) in light blue. 3: Present and interferes with both sense of well‐being and activities of daily living (symptoms), reduced to a level above the ankles (signs) in blue.

Figure 2 presents the frequency of the different symptoms and signs among KTR with and without polyneuropathy. All symptoms and signs were observed more frequently in KTR with polyneuropathy compared to KTR without polyneuropathy (see Appendix E).

KTR with polyneuropathy most frequently reported symptoms of numbness (52.8%; Scores 1, 2, or 3) and tingling sensations (45.1%; Scores 1, 2, or 3) in the feet. Neurological testing revealed frequent abnormalities in nociception (81.3%; Scores 1, 2 or 3) and abnormal light touch perception (48.4%; Scores 1, 2, or 3). In contrast, KTR without polyneuropathy predominantly reported tingling sensations in the feet (15.9%; Score 1) and ataxia (14.5%; Scores 1, 2, or 3). During neurological testing, they most often exhibited abnormal nociception (36.2%; Scores 1, 2, or 3) and abnormal vibration perception (8.7%; Scores 1 or 2).

### 3.3 Validation of the mTCNS

In logistic regression analyses with Firth penalization, the attributions of the mTCNS components were assessed (see Table 2).

**TABLE 2:** Firth‐penalized logistic regression of mTCNS components.

		Univariable logistic regression		Multivariable logistic regression	
		OR (95% CI)	*p*	OR (95% CI)	*p*
	Intercept			0.3 (0.1–0.5)	
Symptoms	Pain feet	3.7 (1.8–11.7)	**< 0.001**	1.4 (0.6–4.6)	0.53
	Numbness feet	8.1 (3.6–25.0)	**< 0.001**	4.9 (1.8–18.0)	**0.001**
	Tingling sensation feet	3.4 (1.9–6.7)	**< 0.001**	2.5 (1.2–5.9)	**0.02**
	Weakness	1.6 (1.0–2.9)	**0.04**	0.7 (0.2–1.6)	0.38
	Ataxia	1.8 (1.2–2.7)	**0.002**	1.1 (0.6–2.2)	0.69
	Upper limb symptoms	1.7 (1.1–2.8)	**0.01**	0.8 (0.3–1.5)	0.43
Signs	Abnormal nociception	2.3 (1.7–3.1)	**< 0.001**	1.5 (1.1–2.2)	**0.02**
	Abnormal light touch perception	3.2 (1.9–6.2)	**< 0.001**	1.4 (0.8–3.1)	0.28
	Abnormal proprioception	10.2 (1.3–1327.3)	**0.02**	0.1 (0.004–6.0)	0.15
	Abnormal vibration perception	2.1 (1.5–3.0)	**< 0.001**	1.5 (1.0–2.4)	**0.04**

*Note:* All mTCNS components were handled as ordinal variables. *p*‐values below 0.05 are considered statistically significant and are highlighted in bold.

Abbreviations: CI, confidence interval based on the profile penalized log likelihood; OR, odds ratio.

In univariable logistic regression analyses, all components of the mTCNS were associated with the presence of polyneuropathy in KTR: pain feet (OR = 3.7 [1.8–11.7]), numbness feet (OR = 8.1 [3.6–25.0]), tingling sensation feet (OR = 3.4 [1.9–6.7]), weakness (OR = 1.6 [1.0–2.9]), ataxia (OR = 1.8 [1.2–2.7]), upper limb symptoms (OR = 1.7 [1.1–2.8]), abnormal nociception (OR = 2.3 [1.7–3.1]), abnormal light touch perception (OR = 3.2 [1.9–6.2]), abnormal proprioception (OR = 10.2 [1.3–1327.3]), and abnormal vibration perception (OR = 2.1 [1.5–3.0]).

In multivariable logistic regression, numbness feet (OR = 4.9 [1.8–18.0]), tingling sensation feet (OR = 2.5 [1.2–5.9]), abnormal nociception (OR = 1.5 [1.1–2.2]), and abnormal vibration perception (OR = 1.5 [1.0–2.4]) remained as significant associations.

### 3.4 Additional Neurological Assessment

In sensitivity analyses, the association with additional neurological assessments, including testing of Achilles tendon reflex, patellar tendon reflex, and muscle strength in lower and upper limbs, was evaluated. The frequency of abnormal findings in these assessments among KTR with and without polyneuropathy is detailed in Appendix E.

In univariable logistic regression analyses, abnormal Achilles tendon reflex (OR = 4.0 [2.6–6.8]), abnormal patellar tendon reflex (OR = 15.5 [3.9–141.0]), and abnormal muscle strength in lower limbs (OR = 3.0 [1.5–6.2]) were associated with the presence of polyneuropathy (see Appendix F and Table F1). In multivariable logistic regression, next to the earlier identified components numbness feet (OR = 5.7 [2.0–22.5]) and tingling sensation feet (OR = 2.5 [1.1–6.8]), abnormal Achilles tendon reflex (OR = 2.5 [1.4–4.8]) and abnormal patellar tendon reflex (OR = 5.8 [1.0–59.8]) remained as significant associations (see Appendix F and Table F2).

### 3.5 Kidney Transplant Neuropathy Score

Following a stepwise recursive elimination procedure, a reduced model of the mTCNS was reached, the KTNS_Basic_. As shown in Table 3, the KTNS_Basic_ entails the components numbness feet (OR = 5.8 [2.2–20.6]), tingling sensation feet (OR = 2.7 [1.3–6.5]), abnormal nociception (OR = 1.7 [1.2–2.4]), and abnormal vibration perception (OR = 1.6 [1.1–2.4]).

**TABLE 3:** KTNS components.

	OR (95% CI)	*p*
KTNS_Basic_		
Intercept	0.2 (0.1–0.4)	
Numbness feet	5.8 (2.2–20.6)	**< 0.001**
Tingling sensation feet	2.7 (1.3–6.5)	**0.01**
Abnormal nociception	1.7 (1.2–2.4)	**0.002**
Abnormal vibration perception	1.6 (1.1–2.4)	**0.03**
KTNS_Advanced_		
Intercept	0.1 (0.1–0.3)	
Numbness feet	8.1 (2.8–29.7)	**< 0.001**
Tingling sensation feet	3.1 (1.4–8.3)	**0.01**
Abnormal nociception	1.6 (1.1–2.4)	**0.01**
Abnormal Achilles tendon reflex	2.4 (1.3–4.4)	**0.004**
Abnormal patellar tendon reflex	8.3 (1.5–87.7)	**0.01**

*Note:* Method: Stepwise recursive elimination procedure. *p*‐values below 0.05 are considered statistically significant and are highlighted in bold.

Abbreviations: CI, confidence interval based on the profile penalized log likelihood; OR, odds ratio.

In sensitivity analyses, the same procedure was applied with inclusion of the additional testing components (i.e., Achilles tendon reflex, patellar tendon reflex, and muscle strength in lower and upper limbs). In the KTNS_Advanced_ numbness feet (OR = 8.1 [2.8–29.7]), tingling sensation feet (OR = 3.1 [1.4–8.3]), abnormal nociception (OR = 1.6 [1.1–2.4]), abnormal Achilles tendon reflex (OR = 2.4 [1.3–4.4]), and abnormal patellar tendon reflex (OR = 8.3 [1.5–87.7]) were included, see Table 3.

**FIGURE 3:**
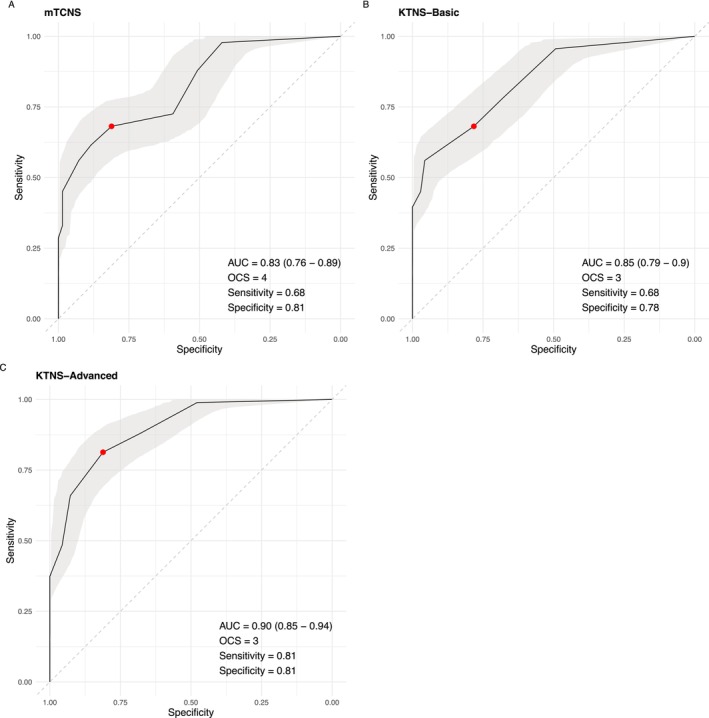
ROC curve with bootstrapped confidence bands for the mTCNS and KTNS. AUC, area under the curve; OCS, optimal cutoff score (red data point in figure, based on Euclidean index); ROC, receiver–operator curve.

Both the mTCNS and KTNS demonstrated good performance, with an AUC of 0.83 (95% CI: 0.76–0.89) for the mTCNS, an AUC of 0.85 (95% CI: 0.79–0.90) for the KTNS_Basic_, and an AUC of 0.90 (95% CI: 0.85–0.94) for the KTNS_Advanced_ (see Figure 3). As shown in Table 4, the observed AUC of the KTNS_Advanced_ was significantly higher than the observed AUC of the mTCNS and of the KTNS_Basic_ (*p*_adj_ = 0.01 and *p*_adj_ = 0.02, resp.), while there was no statistically significant difference between the observed AUCs of the mTCNS and the KTNS_Basic_ (*p*_adj_ = 0.35).

**TABLE 4:** Comparison of AUC of different screening tools.

	ΔAUC^a^	*p*	Adj. *p* *
mTCNS vs. KTNS_Basic_	−0.02	0.35	0.35
mTCNS vs. KTNS_Advanced_	−0.07	**0.003**	**0.01**
KTNS_Advanced_ vs. KTNS_Basic_	0.05	**0.01**	**0.02**

*Note:* *p*‐values below 0.05 are considered statistically significant and are highlighted in bold.

* Holm's correction for multiple testing.

^a^ ΔAUC = AUC (tool A) − AUC (tool B).

**FIGURE G1:**
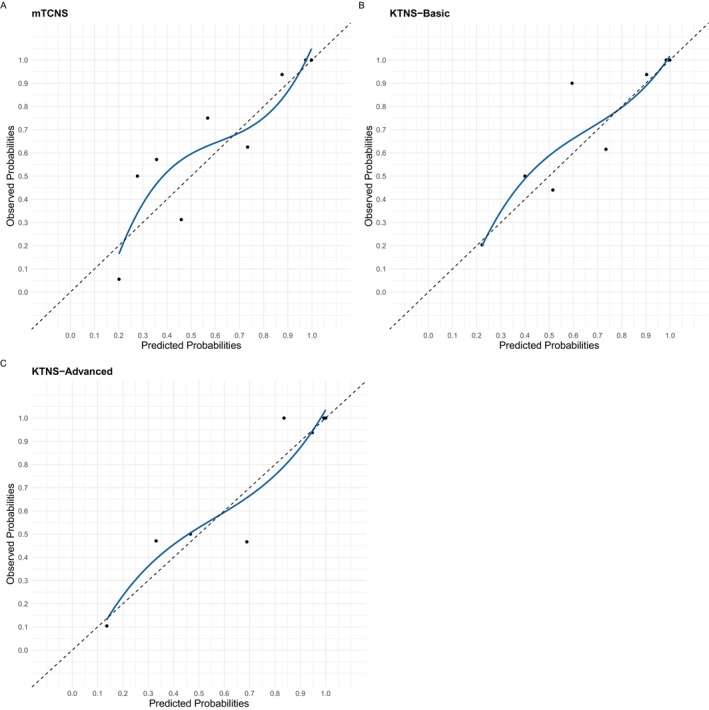
Calibration curves. Agreement between predicted and observed probabilities of logistic regression models depicted as calibration curve for the (A) mTCNS, (B) KTNS_Basic_, and (C) KTNS_Advanced_.

**FIGURE G2:**
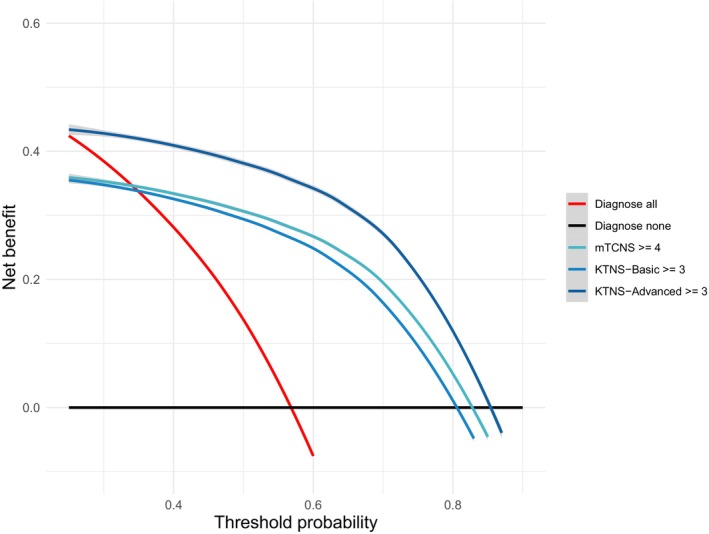
Decision curve analysis. The figure displays the net benefit curves for the mTCNS, KTNS_Basic_ and KTNS_Advanced_. The *X*‐axis shows the threshold probability, which can be regarded as the probability the diagnostic model assigns a diagnosis of polyneuropathy. Differences in net benefit (displayed on the *Y*‐axis) at a given threshold probability between two scores can be interpreted as the increase in the number of true polyneuropathy diagnoses, with the number of false positive diagnoses remaining the same. All presented diagnostic models show a higher net benefit than diagnosing all (red curve), from which we conclude that either diagnostic model would lead to more accurate diagnoses, with the KTNS_Advanced_ and KTNS_Basic_ having the best performance. N.B. The displayed decision curve analysis used the optimal cutoff scores for each model according to the Euclidean Index.

Calibration plots and decision curve analysis are displayed in Appendix G; Figures G1 and G2 and confirm these findings.

### 3.6 Cutoff Scores for Diagnosis of Polyneuropathy

**FIGURE H1:**
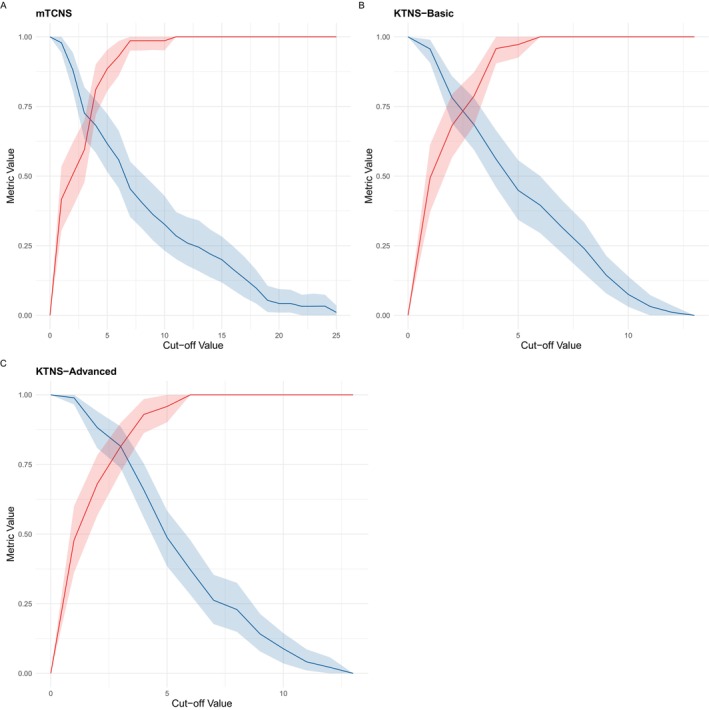
Performance of different cutoff scores of mTCNS and KTNS. This figure illustrates the sensitivity (blue line) and specificity (red line) for different cutoff scores, which are displayed on the *x*‐axis for the (A) mTCNS, (B) KTNS_Basic_, and (C) KTNS_Advanced_.

For use of the scoring systems in clinical practice, sensitivity and specificity of different cutoff scores for the mTCNS, the KTNS_Basic_, and the KTNS_Advanced_ were calculated (Appendix H; Table H1 and Figure H1).

Based on the Euclidian Index, the optimal cutoff score for the mTCNS is ≥ 4 out of a maximum of 30, with a sensitivity of 0.68 and a specificity of 0.81. For the KTNS_Basic_, the optimal cutoff score is ≥ 3 out of a maximum of 12, with a sensitivity of 0.68 and a specificity of 0.87. For the KTNS_Advanced_, the optimal cutoff score is ≥ 3 out of a maximum score of 13, with a sensitivity of 0.81 and a specificity of 0.81.

## 4 Discussion

This validation study on polyneuropathy in KTR identified numbness and tingling in the feet, along with abnormal nociception and vibration perception, as the key components of the mTCNS. The mTCNS demonstrated good performance (AUC = 0.83). To further improve clinical applicability and diagnostic performance, we propose two enhanced versions: the KTNS_Basic_ and the KTNS_Advanced_. The KTNS_Basic_ includes the predominant components, that is, history of numbness and tingling in the feet, abnormal nociception, and abnormal vibration perception; the KTNS_Advanced_ replaces vibration perception testing with Achilles and patellar deep tendon reflex testing. Although both versions showed good diagnostic accuracy, they serve different purposes in clinical practice. The KTNS_Basic_ (AUC = 0.85) performs comparably to the mTCNS while offering a streamlined and less time‐consuming approach that may be suitable for screening by non‐neurologists and in large‐scale research studies, requiring minimal training for reliable application (i.e., a single instruction from a neurologist on handling the instruments and scoring the results). The KTNS_Advanced_ (AUC = 0.90) provides superior diagnostic accuracy but requires more specialized expertise for reflex testing, making it more appropriate for neurological settings or when higher diagnostic certainty is needed before additional testing. The KTNS was internally validated using a bootstrapping method and offers an accurate method for diagnosing polyneuropathy in KTR.

The most common symptoms in KTR with polyneuropathy included numbness and tingling in the feet, both of which were also included in the KTNS. This finding aligns with previous research on chronic polyneuropathy, where these symptoms were the most prevalent and showed high sensitivity for diagnosis [25]. Notably, the validation study of the mTCNS in patients with diabetic sensorimotor polyneuropathy explored symptoms from the patient's perspective, with 100% of participants rating numbness and 90% tingling as important to extremely important [12]. The initial study on the TCNS performed sural nerve biopsies in patients with diabetic neuropathy and found that numbness was associated with fewer axon clusters, underscoring the clinically significant role of axon clusters [11].

Among sensory testing, pinprick testing for nociception was the most frequently abnormal finding in KTR with polyneuropathy and was included in the KTNS. Additionally, the KTNS_Basic_ involves the testing of vibration perception. Existing literature indicates that both pinprick and vibration perception testing are essential for diagnosing polyneuropathy of various etiologies since they significantly contributed to high sensitivity [12, 26]. Pinprick testing assesses both large and small fiber function, relevant since sensor‐predominant and sensorimotor polyneuropathy are the most common types observed in KTR [26, 27]. It is important to note that pinprick testing can be affected by factors such as topographical variations in intraepidermal nerve fiber distribution and regional differences in skin thickness [28, 29]. Vibration perception reflects large fiber function and correlates well with NCS. It is the most common sign in distal symmetric polyneuropathy and chronic inflammatory demyelinating polyneuropathy [26]. Previous studies have identified vibration perception to be the least specific test for polyneuropathy, likely due to age‐related changes in vibration perception [26]. To avoid this bias, we used age‐corrected normal values to determine vibration perception thresholds [15]. Furthermore, in the current study, vibration perception was quantified using a biothesiometer, which has previously been shown to be superior to the tuning fork commonly used in clinical practice [30]. It should be noted that a biothesiometer is not commonly available in everyday clinical practice and was chosen for this study because of its simple handling, its ability to produce quantifiable results, and its low inter‐observer variability [31]. A Rydel–Seiffer tuning fork has been shown to perform similarly regarding reproducibility and as a diagnostic tool in patients with suspected polyneuropathy and may be more widely available [32]. The results of the additional analyses indicated that the testing of deep tendon reflexes would be superior to vibration perception testing. Reduced or absent deep tendon reflexes, that is, Achilles and patellar tendon reflexes, like impaired vibration perception, indicate large fiber abnormalities [33]. Interestingly, in our cohort, the Achilles tendon reflex showed lower specificity for detecting polyneuropathy compared to the patellar tendon reflex, likely due to the influence of age and low muscle mass in KTR on Achilles reflex testing [34, 35, 36, 37]. However, reflex testing was previously removed from the TCNS due to the high inter‐rater variability [12]. Important to note is that in this study, reflexes were assessed rigorously by an experienced researcher or neurologist, decreasing the risk of inter‐rater variability. Thus, the choice of score for diagnosing polyneuropathy in KTR depends on context; if experienced neurologists can examine reflexes, as in this study, reflex testing is preferable to vibration perception. However, for large cohort studies or for use in clinical practice by clinicians of various disciplines, a standardized and easy‐to‐perform method is preferred. Light touch testing, despite showing marked differences between groups (48% abnormal in KTR with polyneuropathy vs. 6% in KTR without polyneuropathy), was not independently associated with polyneuropathy in multivariable analysis. It has indeed been shown to have high specificity but lower sensitivity for polyneuropathy diagnosis [26]. Previous research suggests light touch testing may be more valuable for predicting polyneuropathy development than for diagnosis, though this requires confirmation in KTR populations [38].

These findings have immediate clinical implications for transplant care. The KTNS_Basic_ allows transplant physicians to perform polyneuropathy screening in about 5 min during routine follow‐up visits. In settings with neurological expertise, the KTNS_Advanced_ is estimated to take a comparable amount of time, while the original mTCNS is estimated to take approximately 10 min to complete. These time estimates for administering the KTNS are based on practical clinical experience. The high sensitivity of lower cutoff scores (> 80%) makes the KTNS particularly suitable for initial screening, whereas the specificity at higher cutoff scores (> 85%) aids in determining which patients require further neurophysiological testing. This could enable earlier detection of potentially treatable cases and timely referrals to neurologists. Similar to diabetes care, it creates a focus area that, once risk factors are identified in the future, would provide recommendations like foot care in diabetes.

This is the first study to validate an assessment tool for polyneuropathy in KTR, in a cohort largely representative of the broader KTR population, as demonstrated by a comparison of baseline characteristics of KTR in the SENS study and the TransplantLines Biobank and Cohort Study. To minimize the test review bias, assessors were blinded during the study visit to either the results of NCS and QST or the mTCNS. Additionally, the mTCNS and KTNS components were treated as ordinal variables to obtain the most informative scoring of the signs and symptoms. The methodological strengths of this validation study support the potential of the KTNS to be implemented in clinical practice or large cohort studies, enabling use by physicians in internal medicine and nephrology. The cutoff scores guide further examinations on the etiology and potential neurologist referrals. Depending on the aim and the setting, such as determining the presence of polyneuropathy in a patient with sensory complaints or screening of a population in a research setting, the model's performance will differ, and different cutoff scores may be required. Higher cutoff scores might be used for outpatient settings to enhance specificity, while lower scores may be preferred for population studies to increase sensitivity. Until now, the diagnosis of polyneuropathy remains complex, and eventually an expert opinion will be needed for patients with abnormal KTNS findings [7]. Several methodological considerations are important. First, it is possible that experiencing symptoms of polyneuropathy increased interest in participating in the current study, which could have led to an overestimation of symptom prevalence in our cohort and may limit the generalizability of the findings to the broader KTR population with more asymptomatic individuals. Second, the quasi‐complete separation of proprioception data, while addressed through Firth penalization, limits conclusions about this component. Third, some degree of incorporation bias was unavoidable since symptoms and signs necessarily contribute to both the reference standard and the diagnostic score [39, 40]. Fourth, the small number of patients with small fiber neuropathy in this cohort (*n* = 7) made it impossible to differentiate among affected nerve fiber types and their underlying pathophysiologies for validating the score. Finally, although internal validation through bootstrapping supports the robustness of our findings, external validation in different KTR populations is needed to confirm generalizability. Future research should focus on external validation of the KTNS in different transplant centers and assess its ability to detect changes in severity of polyneuropathy over time. In addition, the next crucial step will be to evaluate the feasibility and diagnostic performance of the KTNS when utilized by non‐neurological experts in clinical practice. This could establish the KTNS as a valuable tool for both clinical monitoring and research applications in the transplant population.

In summary, the mTCNS demonstrated good performance in diagnosing polyneuropathy in KTR. To enhance both diagnostic accuracy and clinical applicability, we propose the KTNS, which includes the following key components: history of numbness and tingling, testing for nociception, and vibration perception or deep tendon reflexes. In the long term, the KTNS has significant potential to assist clinicians from various disciplines without neurological expertise in diagnosing polyneuropathy.

## Conflicts of Interest

The authors declare no conflicts of interest.

## Data Availability

The datasets with individual de‐identified participant data generated during and/or analyzed during the current study are available from the corresponding author upon reasonable request.

## Appendix A Neurological Testing Protocol of the Modified Toronto Clinical Neuropathy Score

As shown in Figure A1, nociception was tested using two neuropens, one with the blunt end of the neurotip extended (1), and the other with the sharp end of the neurotip extended (2). When using the neuropen, the investigator pressed down until the neurotip reached the 40‐g marked area and held it in place for 1–2 s. The participant was pricked five times in total on the big toe with either neurotip in random order. If they failed to distinguish blunt from sharp at least one time, the same procedure was followed on the dorsum of the foot, followed by the shin if the foot was abnormal.

When using the 10‐g monofilament to assess light touch perception (see Figure A2), the investigator pushed it onto the participant's skin until it bowed before holding it in position for 1–2 s. For this test, the monofilament was touched to the participant's skin on either count 1 or count 2. Subsequently, the participant was asked to indicate on which count they felt a touch. The same procedure was carried out in total five times in a random order. If the participant failed at least once to correctly identify on which count the monofilament was touching them, the investigator continued to the dorsum of the foot and potentially the shin.

As illustrated in Figure A3, proprioception was tested by dorsiflexion of the big toe (1) or plantarflexion of the big toe (2), five times in a random order. The big toe was held firmly on the side to not apply pressure on the dorsal or plantar side. The participant was asked to indicate the direction of the movement. If position sense was reduced at the level of the big toe, the investigator continued by moving the entire foot and subsequently the index finger.

Vibration perception was tested using a handheld biothesiometer with an applicator button that vibrates at a frequency of 120 Hz and a vibration amplitude ranging from 0 to 25.5 μm of motion. The following three locations were tested on the patient's body: the tip of the first toe (1), the instep (2) and the lateral malleolus (3) (see Figure A4). Participants were familiarized with the sensation of the biothesiometer by applying it to their hand and increasing the vibration amplitude. Care was taken to rest the applicator on a bony prominence without applying any pressure apart from the weight of the applicator. The vibration amplitude was slowly increased to a point at which the participant could perceive it. All locations were measured twice, with a third measurement being performed if the difference between the first two measurements was > 20%.

Sensory test scores were graded as 1 if sensation was reduced at both toes only, 2 if it was additionally reduced on both sides at the instep, and 3 if it was further reduced to a level above the lateral malleoli at both sides. Otherwise, they were graded as 0.

Testing of temperature sense was omitted due to logistical constraints.

## Appendix B Protocol Staged Approach for the Classification of Polyneuropathy

Polyneuropathy was classified based on the neurological screening and divided by the type of nerve fiber affected. During the classification process, information on an earlier polyneuropathy diagnosis was not considered and was unavailable to the expert panel.

The following criteria were used for **large fiber polyneuropathy**:

Based on the following three screening components, KTR were classified: Presentation with symptoms, abnormal neurological examination, and abnormal NCS.

– “No” polyneuropathy: no abnormalities in all screening components
– “Possible” polyneuropathy: abnormalities in one screening component
– “Probable” polyneuropathy: abnormalities in at least two screening components, of which the participant had to exhibit at least one abnormal NCS
– “Definite” polyneuropathy: abnormalities in all screening components

The following criteria were used for **small fiber neuropathy**:

Based on the following abnormalities in the neurological screening, small fiber polyneuropathy was excluded: abnormal reflexes, abnormal vibration perception or proprioception, and abnormalities in NCS.

– “Probable” small fiber neuropathy: abnormalities in all screening components (i.e., symptoms typical for small fiber neuropathy such as allodynia, tingling or numbness, abnormal nociception testing and QST)
– A skin biopsy, essential for meeting the diagnostic criteria of definite small fiber neuropathy, was not performed as it was not included in the study protocol.

### Decisions Taken During the Screening Process as Part of the Plenary Panel

Most decisions are based on the overall goal of prioritizing specificity over sensitivity to minimize the number of false positives.

– Abnormalities in **anamnesis**:
  ○ In case of participants reporting exclusively abnormalities at the hands, NCS and QST outcomes at the hands' level were considered.
  ○ In case of participants reporting exclusively symptoms of ataxia or weakness, outcomes of NCS and QST were taken into consideration.
– **Neurological examination**:
  ○ In the event of a discrepancy between nociception and light touch testing, an abnormality in either test is regarded as a valid indication of an abnormal neurological examination.
  ○ Participants showed a high prevalence of abnormal muscle strength testing, but in most cases not according to the typical polyneuropathy distribution. Muscle strength measurement was taken into consideration in cases of serious sensorimotor polyneuropathy.
  ○ In the event of asymmetrical reflex testing results, the most normal score was used for the evaluation.
  ○ A reduced or absent Achilles tendon reflex was considered significant only when accompanied by an absent soleus H‐reflex.
– **NCS**:
  ○ In cases of discrepancy between the peroneal and tibial nerves, the higher score of the two was applied.
  ○ In the case of mild abnormalities of the sural nerve amplitude around the age of 60, the results of the sensory ulnar nerve were taken into consideration. With caution, these results were scored as abnormal.
  ○ In case of slightly decreased nerve conduction velocities, skin temperature and the results of other NCS elements were taken into consideration.

## Appendix C Comparison of Baseline Characteristics Between SENS Study Cohort and TransplantLines Cohort

**Table:**

	Cohort SENS study	Cohort TransplantLines	*p*
*N*	160	1473	
Age, years	59.8 ± 11.6	57.8 ± 14.0	**0.04**
Sex, *n* (%) male	107 (66.9)	893 (60.6)	0.15
Primary disease^a^, *n* (%)			
Unknown	26 (16.3)	240 (16.3)	0.24
Glomerulonephritis	45 (28.1)	327 (22.2)	
Pyelonephritis	13 (8.1)	139 (9.4)	
Cystic kidney disease	37 (23.1)	277 (18.8)	
Other congenital/hereditary disease	5 (3.1)	60 (4.1)	
Hypertensive nephropathy	14 (8.8)	163 (11.1)	
Diabetic nephropathy	11 (6.9)	109 (7.4)	
Other multisystem diseases	7 (4.4)	65 (4.4)	
Other	2 (1.3)	62 (4.2)	
Diabetes^b^, *n* (%)	36 (22.5)	348 (25.2)	0.51
Alcohol intake^c^, units/week	1.2 [0–6.3]	0.6 [0–5.4]	0.44
Smoking status^d^, *n* (%)			
Never	79 (54.5)	495 (45.8)	0.11
Ex‐smoker	57 (39.3)	484 (44.8)	
Current	9 (6.2)	102 (9.4)	
Immunosuppression			
Calcineurin inhibitor use^e^, *n* (%)	137 (85.6)	1214 (88.0)	0.45
Tacrolimus use, *n* (%)	118 (73.8)	1051 (76.2)	0.55
Cyclosporine use, *n* (%)	19 (11.9)	166 (12.0)	0.99

*Note:* Missing data (*n*): ^a^TransplantLines: 31; ^b^TransplantLines: 94; ^c^SENS: 12, TransplantLines: 474; ^d^SENS: 15, TransplantLines: 392; ^e^TransplantLines: 94. *p*‐values below 0.05 are considered statistically significant and are highlighted in bold.

## Appendix D Comparison of Baseline Characteristics Between KTR With and Without Polyneuropathy

**Table:**

	PNP^a^	No PNP	*p*
*N*	91	69	
Age, years	62.7 ± 10.4	56.7 ± 12.2	**< 0.001**
Sex, *n* (%) male	71 (78.0)	36 (52.2)	**0.001**
Height, cm	178.0 ± 8.3	172.7 ± 9.0	**0.002**
Weight, kg	84.9 ± 14.5	76.6 ± 12.5	**< 0.001**
Time since transplantation, years	6.6 [3.6–12.5]	5.3 [4.0–14.2]	0.88
Primary disease, *n* (%)			
Unknown	10 (11.9)	14 (20.3)	0.41
Glomerulonephritis	26 (31.0)	18 (26.1)	
Pyelonephritis	7 (8.3)	6 (8.7)	
Cystic kidney disease	18 (21.4)	17 (24.6)	
Other congenital/hereditary disease	2 (2.4)	3 (4.4)	
Hypertensive nephropathy	7 (8.3)	7 (10.1)	
Diabetic nephropathy	9 (10.7)	1 (1.5)	
Other multisystem diseases	4 (4.8)	2 (2.9)	
Other	1 (1.2)	1 (1.5)	
Diabetes, *n* (%)	26 (28.6)	10 (14.5)	0.06
Type 1 diabetes	5 (5.5)	0	
Type 2 diabetes	10 (11.0)	2 (2.9)	
PTDM	11 (12.1)	8 (11.6)	
Pre‐emptive transplantation, *n* (%)	32 (35.7)	35 (50.7)	0.07
Dialysis prior to transplantation, *n* (%)	58 (64.4)	34 (49.3)	0.08
Hemodialysis	41 (45.1)	14 (20.3)	
Peritoneal dialysis	24 (26.4)	22 (31.9)	
Dialysis vintage, months	25 [9–48]	23 [9–37]	0.66
Type of transplantation			
Kidney	87 (95.6)	69 (100.0)	0.21
Combined kidney and pancreas	4 (4.4)	0	
Donor type, *n* (%)			
Living donor	51 (56.0)	46 (66.7)	0.23
Deceased donor	40 (44.0)	23 (33.3)	
Donation after brain death	21 (52.5)	14 (63.6)	
Donation after circulatory death	19 (47.5)	8 (36.4)	
Alcohol intake, units/week	0.8 [0–6.2]	1.3 [0–6.5]	0.59
Smoking status, *n* (%)			
Never	35 (44.9)	44 (65.7)	**0.04**
Ex‐smoker	38 (48.7)	19 (28.4)	
Current	5 (6.4)	4 (6.0)	
Immunosuppression			
Calcineurin inhibitor use, *n* (%)	80 (87.9)	57 (82.6)	0.47
Tacrolimus use, *n* (%)	65 (71.4)	53 (76.8)	0.56
Tacrolimus dosage, mg	3.0 [1.9–4.0]	3.0 [2.0–4.0]	0.97
Whole blood trough concentration tacrolimus, μg/L	5.3 ± 1.6	4.9 ± 1.2	0.15
Cyclosporine use, *n* (%)	15 (16.5)	4 (5.8)	0.07
Cyclosporine dosage, mg	153.3 ± 66.7	187.5 ± 47.9	0.36
Whole blood trough concentration cyclosporine, μg/L	68.0 [49.0–97.5]	80.0 [77.3–86.5]	0.89
Prednisolone use, *n* (%)	90 (98.9)	65 (94.2)	0.22
Third immunosuppressive			
MMF, *n* (%)	68 (74.7)	53 (76.8)	0.86
Everolimus, *n* (%)	7 (7.7)	3 (4.4)	
Azathioprine, *n* (%)	4 (4.4)	3 (4.4)	
Other, *n* (%)	3 (3.3)	1 (1.4)	

*Note:* *p*‐values below 0.05 are considered statistically significant and are highlighted in bold.

Abbreviations: MMF, mycophenolate mofetil; PTDM, post‐transplant diabetes mellitus.

^a^Including KTR with large fiber polyneuropathy and small fiber neuropathy.

## Appendix E Comparison of Frequency of Symptoms and Signs Between KTR With and Without Polyneuropathy

**Table:**

	KTR with PNP, *n* (%)	KTR without PNP, *n* (%)	*p*
Modified Toronto Clinical Neuropathy Score			
Pain feet	24 (26.4)	3 (4.4)	**< 0.001**
Numbness feet	48 (52.8)	4 (5.8)	**< 0.001**
Tingling sensation feet	41 (45.1)	11 (15.9)	**< 0.001**
Weakness	17 (18.7)	5 (7.3)	0.06
Ataxia	34 (37.4)	10 (14.5)	**0.002**
Upper limb symptoms	31 (34.1)	9 (13.0)	**0.004**
Abnormal nociception	74 (81.3)	25 (36.2)	**< 0.001**
Abnormal light touch perception	44 (48.4)	4 (5.8)	**< 0.001**
Abnormal proprioception	7 (7.7)	0 (0)	0.05
Abnormal vibration perception	40 (44.0)	6 (8.7)	**< 0.001**
Additional neurological assessment			
Abnormal Achilles tendon reflex	61 (67.0)	12 (17.4)	**< 0.001**
Abnormal patellar tendon reflex	25 (27.5)	1 (1.5)	**< 0.001**
Abnormal muscle strength lower limbs	75 (82.4)	42 (60.9)	**0.004**
Abnormal muscle strength upper limbs	67 (73.6)	41 (59.4)	0.08

*Note:* *p*‐values below 0.05 are considered statistically significant and are highlighted in bold.

Abbreviations: KTR, kidney transplant recipients; PNP, polyneuropathy.

## Appendix F Assessment of Additional Neurological Assessments (i.e., Reflexes and Muscle Strength)

**TABLE F1:** Univariable logistic regression—additional neurological assessment components.

	OR (95% CI)	*p*
Abnormal Achilles tendon reflex	4.0 (2.6–6.8)	**< 0.001**
Abnormal patellar tendon reflex	15.5 (3.9–141.0)	**< 0.001**
Abnormal muscle strength lower limbs	3.0 (1.5–6.2)	**0.003**
Abnormal muscle strength upper limbs	1.9 (1.0–3.7)	0.06

*Note:* Achilles and patellar tendon reflexes were handled as ordinal variables, muscle strength was included as dichotomous variable (normal vs. abnormal). *p*‐values below 0.05 are considered statistically significant and are highlighted in bold.

Abbreviations: CI, confidence interval based on the profile penalized log likelihood; OR, odds ratio.

**TABLE F2:** Multivariable logistic regression including additional neurological assessment components.

	OR (95% CI)	*p*
Intercept	0.1 (0.04–0.3)	
Pain feet	1.7 (0.6–6.4)	0.31
Numbness feet	5.5 (1.9–21.2)	**0.001**
Tingling sensation feet	2.5 (1.1–6.8)	**0.03**
Weakness	0.7 (0.2–1.6)	0.36
Ataxia	1.1 (0.6–2.3)	0.74
Upper limb symptoms	0.8 (0.4–1.7)	0.61
Abnormal nociception	1.3 (0.9–2.0)	0.16
Abnormal light touch perception	1.5 (0.8–3.0)	0.22
Abnormal proprioception	0.1 (0.003–5.0)	0.14
Abnormal vibration perception	1.2 (0.8–2.1)	0.41
Abnormal Achilles tendon reflex	2.5 (1.4–4.8)	**0.002**
Abnormal patellar tendon reflex	5.8 (1.0–59.8)	**0.05**
Abnormal muscle strength lower limbs	1.7 (0.5–6.0)	0.36
Abnormal muscle strength upper limbs	0.8 (0.2–2.4)	0.62

*Note:* All mTCNS components, Achilles and patellar tendon reflexes were handled as ordinal variables, muscle strength was included as dichotomous variable (normal vs. abnormal). *p*‐values below 0.05 are considered statistically significant and are highlighted in bold.

Abbreviations: CI, confidence interval based on the profile penalized log likelihood; OR, odds ratio; SE: standard error of the logits (log‐odds).

## Appendix H Cutoff Scores for Diagnosis of Polyneuropathy

**TABLE H1:** Bootstrapped performance metrics.

Cutoff score	Sensitivity (95% CI)	Specificity (95% CI)	Euclidean index
mTCNS			
1	0.98 (0.95–1.00)	0.42 (0.30–0.54)	0.58
2	0.88 (0.81–0.94)	0.51 (0.39–0.62)	0.51
3	0.72 (0.63–0.82)	0.59 (0.46–0.70)	0.49
4	0.69 (0.58–0.78)	0.81 (0.71–0.90)	**0.37**
5	0.62 (0.52–0.72)	0.89 (0.81–0.95)	0.40
KTNS_Basic_			
1	0.96 (0.91–0.99)	0.49 (0.38–0.62)	0.51
2	0.78 (0.70–0.86)	0.69 (0.57–0.79)	0.39
3	0.68 (0.59–0.78)	0.79 (0.68–0.87)	**0.38**
4	0.56 (0.46–0.66)	0.96 (0.91–1.00)	0.44
5	0.45 (0.35–0.55)	0.97 (0.93–1.00)	0.55
KTNS_Advanced_			
1	0.99 (0.96–1.00)	0.48 (0.35–0.59)	0.53
2	0.88 (0.81–0.94)	0.69 (0.57–0.79)	0.34
3	0.82 (0.73–0.89)	0.82 (0.71–0.90)	**0.27**
4	0.66 (0.57–0.76)	0.93 (0.86–0.99)	0.35
5	0.49 (0.38–0.59)	0.96 (0.91–1.00)	0.51

*Note:* Bootstrapped approach for performance metrics (sensitivity and specificity) with 1000 resamples for internal validation. Euclidean index = $\sqrt{{\left(1-\text{sensitivity}\right)}^{2}+{\left(1-\text{specificity}\right)}^{2}}$. *p*‐values below 0.05 are considered statistically significant and are highlighted in bold.

Abbreviations: CI, confidence interval; KTNS, Kidney Transplant Neuropathy Score; mTCNS, modified Toronto Clinical Neuropathy Score.
